# Activation of aryl hydrocarbon receptor by cigarette smoke induces oxidative stress, autophagy impairment and cellular senescence

**DOI:** 10.18332/tid/226746

**Published:** 2026-09-09

**Authors:** Bao-Yu Shi, Xu-Dong Hu, Yuan-Cheng Liu, Si-Ming Hu, Guo-Xing Zhang

**Affiliations:** 1 Department of Respiratory MedicineThe Affiliated Suzhou Hospital of Nanjing Medical University, Suzhou Municipal Hospital, Gusu School of Nanjing Medical UniversitySuzhouChina; 2 Department of Physiology and NeuroscienceSchool of Basic Medical Science, Soochow UniversitySuzhouChina

**Keywords:** chronic obstructive pulmonary disease, aryl hydrocarbon receptor, oxidative stress, autophagy, cellular senescence

## Abstract

**Introduction:**

Cellular senescence is a core pathogenic mechanism of chronic obstructive pulmonary disease (COPD), and cigarette smoke (CS) acts as its primary risk factor. However, the molecular cascade linking CS exposure to pulmonary cellular senescence remains poorly defined. The aryl hydrocarbon receptor (AhR), a ligand-activated transcription factor triggered by CS-derived chemicals, has an unclarified function in smoke-induced lung cell senescence. This study aimed to verify whether AhR mediates CS-elicited pulmonary senescence through regulating oxidative stress and autophagy, and to elucidate the complete downstream signaling cascade.

**Methods:**

We established an 8-week whole-body CS exposure rat COPD model and treated human bronchial epithelial BEAS-2B cells with cigarette smoke extract (CSE). Multiple detection approaches were applied to assess lung pathological lesions, cellular senescence, autophagic flux and intracellular reactive oxygen species (ROS). We further performed transcriptomic sequencing on rat lung tissues and mined three public human COPD Gene Expression Omnibus (GEO) datasets to screen key differential genes. Rescue experiments with rapamycin (autophagy activator), N-acetylcysteine (NAC, ROS scavenger) and CH223191 (specific AhR antagonist) were conducted to validate causal relationships among AhR, ROS, autophagy and senescence.

**Results:**

*In vivo* CS exposure induced typical COPD-like pathological alterations and prominent cellular senescence in rat lungs. *In vitro* CSE treatment triggered dose-dependent senescence in BEAS-2B cells, accompanied by impaired autophagic flux and excessive ROS overproduction; rapamycin and NAC separately reversed these two phenotypes. Both *in vivo* and *in vitro* data confirmed robust activation of the AhR pathway, evidenced by significant upregulation of AhR target genes *CYP1A1* and *CYP1B1*. Pharmacological inhibition of AhR via CH223191 suppressed ROS accumulation, restored defective autophagy, and ultimately alleviated CSE-induced cellular senescence.

**Conclusions:**

CS activates pulmonary AhR signaling, which sequentially drives excessive ROS generation, autophagic flux impairment and irreversible lung epithelial senescence. The AhR–ROS–autophagy axis represents a novel pathogenic pathway mediating smoke-related lung aging.

## Introduction

Chronic obstructive pulmonary disease (COPD) is a prevalent global chronic respiratory disease with persistent irreversible airflow limitation, which imposes severe social and economic burdens worldwide^1^. Cigarette smoke (CS) exposure is the predominant risk factor, contributing to more than 80% of all COPD cases^2^. Current clinical interventions only relieve symptoms via anti-inflammatory agents and bronchodilators, lacking effective treatments to halt disease progression or repair damaged lung tissue^3^. Unraveling the molecular mechanisms driving CS-induced pulmonary injury is critical for developing novel targeted COPD therapeutics^4^.

Cellular senescence, defined as irreversible cell cycle arrest accompanied by the senescence-associated secretory phenotype (SASP), acts as a core pathogenic driver of COPD^5,6^. Lungs from COPD patients exhibit robust aging hallmarks, including elevated senescence markers p53, p21 and p16, telomere attrition, and abundant senescent cell accumulation^7,8^. Preclinical animal models confirm that selective elimination of senescent cells alleviates CS-triggered emphysematous lesions, while pharmacological suppression of senescence preserves lung function and mitigates tissue destruction^9,10^, marking cellular senescence as a promising therapeutic target for COPD.

Autophagy represents an intracellular homeostatic catabolic pathway that clears impaired organelles and misfolded proteins^11^. Defective autophagic flux (rather than suppressed autophagy initiation) is a signature feature of CS-mediated lung injury, characterized by massive buildup of the autophagy substrate p62 in COPD lung parenchyma^12,13^. Dysfunctional autophagy leads to toxic cellular debris accumulation, which further amplifies oxidative stress and accelerates cellular senescence^14^. Consistently, rapamycin-mediated autophagy restoration attenuates CS-induced lung damage and senescence^15^, verifying autophagy’s central role in COPD pathogenesis.

Oxidative stress serves as a well-established intermediate mediator linking CS exposure and autophagy dysfunction^12^. CS contains abundant reactive oxygen species (ROS) and pro-oxidant chemicals that drive excessive intracellular ROS generation and deplete endogenous antioxidant defenses in airway epithelial cells^13^. Surplus ROS directly induces cellular damage and disrupts lysosomal-dependent autophagic degradation, resulting in senescent cell accumulation^12^. The ROS scavenger N-acetylcysteine (NAC) effectively rescues CS-impaired autophagic flux and alleviates lung injury, confirming oxidative stress as a vital bridge between CS exposure and autophagy impairment in COPD^12^.

The aryl hydrocarbon receptor (AhR) is a ligand-activated transcription factor highly enriched in pulmonary epithelial cells^16^. Polycyclic aromatic hydrocarbons and quinones within cigarette smoke, function as potent AhR agonists, triggering robust AhR signaling activation both *in vitro* and *in vivo*^16^. Upon activation, AhR upregulates phase I metabolic enzymes CYP1A1 and CYP1B1; sustained excessive AhR activation causes inefficient CYP catalytic cycling, which further boosts ROS overproduction and exacerbates oxidative stress^17^. Prior studies report that AhR knockout aggravates CS-induced pulmonary inflammation and oxidative stress^18,19^, yet the exact regulatory role of AhR in CS-driven cellular senescence, as well as its molecular crosstalk with ROS and autophagy cascades, remains largely uncharacterized^20,21^.

In this study, we combined a CS-induced rat COPD model, CSE-stimulated BEAS-2B cells, and clinical transcriptomic data analysis to investigate the role of AhR in CS-induced pulmonary cellular senescence. We further explored whether AhR mediates senescence via regulating the ROS-autophagy axis, and validated the therapeutic potential of targeting AhR for CS-induced lung aging.

## Methods

### Animals and CS exposure

Specific pathogen-free 8-week-old male Wistar rats (220–250 g) were purchased from Zhejiang Vital River Laboratory Animal Technology Co., Ltd. (Hangzhou, China). Sample selection inclusion criteria were: healthy male rats aged 8 weeks with body weight ranging from 220 g to 250 g, no abnormal respiratory behavior, and no skin lesions. Exclusion criteria were: rats showing continuous cough, weight loss over 10% within the 7-day adaptive feeding period, and suspected bacterial/fungal infection. Rats were housed in the Laboratory Animal Center of Soochow University under specific pathogen-free conditions (22 ± 2°C, 50 ± 5% humidity, 12 hours light/dark cycle, ad libitum food and water). After 7 days of adaptive feeding, rats were randomly divided into control group (filtered air exposure) and CS group (cigarette smoke exposure), with 6 rats per group. A rat model of COPD was established using a whole-body CS exposure system (Yuyan Instrument, Shanghai, China). For CS exposure, rats were placed in a 25 L exposure chamber, and filter-removed Hongqiqu cigarettes (10 mg tar, 0.8 mg nicotine per cigarette, Henan, China) were burned using a smoke generator. The intake pump flow rate was set at 5 L/min, cigarette smoke intake at 25 L/h, and exhaust pump flow rate at 6 L/min to maintain slight negative pressure in the chamber. Each cigarette was burned for ~5 minute, with 6 cigarettes per exposure session. Rats were exposed to CS for 0.5 hour per session, 2 sessions per day, 7 days per week, for a total of 8 weeks. Control rats were exposed to filtered air under the same conditions. All animal experiments were approved by the Institutional Animal Care and Use Committee of Soochow University (Approval No.: SYXK 2023–0021) and conducted in accordance with the Guide for the Care and Use of Laboratory Animals.

### Cigarette smoke extract (CSE) preparation and cell culture

CSE was prepared as previously described with minor modifications^22,23^. Filter-removed Hongqiqu cigarettes were burned, and the smoke was bubbled into 10 mL of serum-free Dulbecco’s Modified Eagle Medium (DMEM, Gibco, USA) at a rate of 1 cigarette every 1.5 minute. The resulting CSE was filtered through a 0.22 µm sterile filter to remove particulates and bacteria. The optical density (OD) of CSE was measured at 320 nm using a microplate reader (Bio-Tek, USA), and the stock solution was adjusted to OD320=0.85 (defined as 100% CSE). Working concentrations of CSE (1%, 2%, 3%) were freshly diluted with complete medium before each experiment.

The human bronchial epithelial BEAS-2B cell line was purchased from Hunan Fenghui Biotechnology Co., Ltd. (Changsha, China) and cultured in high-glucose DMEM supplemented with 10% fetal bovine serum (FBS, Gibco, USA) and 1% penicillin-streptomycin (Gibco, USA) at 37°C in a 5% CO_2_ incubator. Cells were seeded in 6-well, 12-well, or 96-well plates and cultured to ~50% confluence before treatment. Prior to stimulation, cells were serum-starved for 12 hours (DMEM +1% FBS+1% penicillin-streptomycin) to synchronize the cell cycle. Cells were then treated with 0%, 1%, 2%, or 3% CSE for 24 hours for dose-response experiments. For rescue experiments, cells were pre-treated with rapamycin (10 µM, MCE, USA), NAC (5 mM, MCE, USA), or CH223191 (10 µM, MCE, USA) for 1 hour, followed by co-treatment with 2% CSE for an additional 24 hours.

### Hematoxylin-eosin (HE) and periodic acid-schiff (PAS) staining

Rat lung tissues were fixed in 4% paraformaldehyde (Biosharp, China) for 24 hours, embedded in paraffin, and sectioned into 5 µm thick slices. For HE staining, sections were deparaffinized with xylene, rehydrated with a graded ethanol series, stained with hematoxylin for 2 minutes, differentiated with 1% acid alcohol, rinsed with running tap water, and counterstained with eosin for 2 minutes. For PAS staining, deparaffinized and rehydrated sections were oxidized with 1% periodic acid for 10 minutes, rinsed with distilled water, incubated with Schiff reagent for 15 minutes, and counterstained with hematoxylin for 1–2 minute. All sections were dehydrated with ethanol, cleared with xylene, and mounted with neutral balsam. Images were captured under a light microscope (Nikon, Japan), and alveolar wall thickness and the percentage of PAS-positive goblet cells were quantified using ImageJ software (NIH, USA) with at least 5 random fields per section.

Periodic Acid-Schiff (PAS) staining was performed to assess goblet cell hyperplasia and mucus production in rat airway tissues. Briefly, paraffin-embedded lung sections were deparaffinized and rehydrated. After oxidation using periodic acid solution, sections were incubated with Schiff reagent, followed by hematoxylin counterstaining. Images were acquired with an optical microscope. PAS-positive goblet cells residing within the bronchial airway epithelium were quantified, and the proportion of PAS-positive stained area was calculated to evaluate the severity of airway mucus hypersecretion.

### Bronchoalveolar lavage fluid (BALF) collection and cell counting

After 8 weeks of CS exposure, rats were anesthetized with pentobarbital sodium (50 mg/kg, intraperitoneal injection). The trachea was intubated, and the left bronchus was cannulated for BALF collection with 2 mL of ice-cold phosphate-buffered saline (PBS) for 4–5 washes. BALF was collected with a recovery rate >80%, and centrifuged at 300 g for 10 minutes at 4°C. The cell pellet was resuspended in PBS, and total cell counts were determined using a hemocytometer under an inverted microscope after 10-fold dilution.

### Western blotting (WB)

Rat lung tissues or BEAS-2B cells were lysed in ice-cold RIPA lysis buffer (Beyotime, China) supplemented with protease inhibitor cocktail (TargetMol, USA) and phosphatase inhibitor cocktail (TargetMol, USA). Lysates were incubated on ice for 30 minutes and centrifuged at 12000 g for 20 minutes at 4°C. The supernatant was collected as total protein, and protein concentration was quantified using the BCA Protein Assay Kit (Beyotime, China). Equal amounts of protein (30 µg per lane) were separated by 10–12% sodium dodecyl sulfate-polyacrylamide gel electrophoresis (SDS-PAGE) and transferred onto 0.45 µm polyvinylidene fluoride (PVDF) membranes (Millipore, USA). Membranes were blocked with 5% non-fat milk in TBST (Tris-buffered saline with 0.1% Tween-20) for 1 hour at room temperature, then incubated overnight at 4°C with primary antibodies: anti-p53 (1:200, Immunoway, USA), anti-p21 (1:1000, Zenbio, China), anti-LC3 (1:1000, CST, USA), anti-p62 (1:1000, Zenbio, China), anti-CYP1A1 (1:1000, Abcam, UK), anti-CYP1B1 (1:1000, Abcam, UK), and anti-GAPDH (1:5000, Proteintech, China) (loading control). Membranes were washed three times with TBST and incubated with horseradish peroxidase (HRP)-conjugated secondary antibodies (1:5000, Fdbio, China) for 1 hour at room temperature. Immunoreactive bands were visualized using an enhanced chemiluminescence (ECL) substrate (Epizyme, China) and captured with a chemiluminescence imaging system (Tanon, China). Band intensities were quantified using ImageJ software, and target protein expression was normalized to GAPDH.

### Immunohistochemistry (IHC)

Rat lung paraffin sections (5 µm) were deparaffinized, rehydrated, and subjected to antigen retrieval by heating in citrate buffer (pH 6.0) at 95°C for 30 minutes. Endogenous peroxidase activity was blocked with 3% H_2_O_2_ for 15 minutes at room temperature, and non-specific binding was blocked with 3% bovine serum albumin (BSA) for 30 minutes at room temperature. Sections were incubated overnight at 4°C with primary antibodies against p53 (1:200, Immunoway, USA) and p21 (1:200, Zenbio, China), then washed three times with PBS and incubated with HRP-conjugated secondary antibodies (1:500, Fdbio, China) for 1 hour at room temperature. Immunoreactivity was visualized with 3,3’-diaminobenzidine (DAB) substrate (ZSGB-BIO, China), and nuclei were counterstained with hematoxylin (Solarbio, China). Sections were dehydrated, cleared, and mounted, and images were captured under a bright-field microscope (Nikon, Japan). The integrated optical density (IOD) of positive staining was quantified using ImageJ software with at least 5 random fields per section.

### Senescence-associated β-galactosidase (SA-β-gal) staining

SA-β-gal staining was performed using a commercial kit (Beyotime, China) according to the manufacturer’s instructions. BEAS-2B cells were seeded in 12-well plates and treated as indicated. After treatment, cells were washed twice with PBS, fixed with fixative solution for 15 minutes at room temperature, and incubated with SA-β-gal staining solution (pH 6.0) at 37°C in a CO_2_-free incubator for 48 hours. Stained cells were observed under an inverted microscope (Nikon, Japan), and the percentage of SA-β-gal-positive cells (blue-stained) was calculated by counting positive cells and total cells in at least 6 random fields per well.

### Immunofluorescence (IF)

BEAS-2B cells were seeded on glass coverslips in 12-well plates and treated as indicated. Cells were washed twice with PBS, fixed with 4% paraformaldehyde for 20 minutes at room temperature, and permeabilized with 0.2% Triton X-100 (Beyotime, China) for 15 minutes at room temperature. Non-specific binding was blocked with blocking buffer (5% normal goat serum +1% BSA in PBS) for 30 minutes at room temperature. Cells were incubated overnight at 4°C with anti-p21 primary antibody (1:200, Zenbio, China), then washed three times with PBS and incubated with Alexa Fluor 594-conjugated secondary antibody (1:500, Abways, China) for 1 hour at room temperature in the dark. Nuclei were counterstained with DAPI (Beyotime, China) for 5 minutes. Coverslips were mounted onto glass slides with anti-fade mounting medium, and images were captured using a confocal laser scanning microscope (Leica, Germany). The mean fluorescence intensity (MFI) of p21 was quantified using ImageJ software with at least 5 random fields per coverslip.

### Intracellular ROS detection

Intracellular ROS levels were measured using the 2’,7’-dichlorodihydrofluorescein diacetate (DCFH-DA) fluorescence probe (Beyotime, China). BEAS-2B cells were seeded in 96-well plates and treated as indicated. After treatment, cells were incubated with 10 µM DCFH-DA (diluted 1:1,000 in PBS) for 20 minutes at 37°C in the dark. Cells were washed three times with PBS to remove excess probe, and fluorescence images were captured under a fluorescence microscope (Nikon, Japan). Fluorescence intensity was quantified using a microplate reader (Bio-Tek, USA) at an excitation wavelength of 488 nm and emission wavelength of 525 nm, and normalized to the control group.

### Quantitative real-time PCR (qRT-PCR)

Total RNA was extracted from rat lung tissues and BEAS-2B cells using TRIzol reagent (TIANGEN, China) according to the manufacturer’s instructions. RNA concentration and purity were assessed using a NanoDrop spectrophotometer (Thermo Fisher, USA). A total of 1 µg of RNA was reverse-transcribed into cDNA using the HiScript II RT SuperMix (Vazyme, China). qRT-PCR was performed on a Bio-Rad CFX96 Real-Time PCR System (Bio-Rad, USA) using ChamQ SYBR qPCR Master Mix (Vazyme, China). The reaction conditions were: 95°C for 30 seconds, followed by 40 cycles of 95°C for 10 seconds and 60°C for 30 seconds. Gene-specific primers were designed using Primer-BLAST (NCBI) and synthesized by Tsingke Biotechnology Co., Ltd. (Nanjing, China). Glyceraldehyde-3-phosphate dehydrogenase (GAPDH) was used as the reference gene, and relative gene expression was calculated using the 2^−ΔΔCt^ method.

### Transcriptomic analysis and gene expression omnibus (GEO) dataset mining

Rat lung tissues from the control and CS groups (2 rats per group) were used for transcriptomic sequencing by Personal Biotechnology Co., Ltd. (Shanghai, China). Raw sequencing data were filtered, and differentially expressed genes (DEGs) were identified with the criteria of p<0.05 and |fold change (FC)|>1.2. Public clinical COPD datasets (GSE10006, GSE11784, GSE20257) were retrieved from the NCBI GEO database (https://www.ncbi.nlm.nih.gov/geo/). Raw expression data were preprocessed: probe IDs were mapped to gene symbols, outlier values were replaced with group medians, and values <1 were floored to 1. All expression values were log2-transformed, and DEGs were identified with the same criteria (p<0.05, |FC|>1.2). Overlapping DEGs between rat RNA-seq data and human GEO datasets were identified using Venn diagrams, and visualized with heatmaps and volcano plots using the online bioinformatics platform SangerBox (www.sangerbox.com).

### Statistical analysis

All experimental data are presented as mean ± standard error of the mean (SEM) from at least three independent experiments. Statistical analysis was performed using GraphPad Prism 8.0 software (GraphPad Inc., USA). Comparisons between two groups were analyzed using Student’s t-test, and comparisons among multiple groups were analyzed using one-way analysis of variance (ANOVA) followed by Tukey’s *post hoc* test. A p<0.05 was considered statistically significant (^*^p<0.05, ^**^p<0.01, ^***^p<0.001 vs control group; ^#^p<0.05, ^##^p<0.01, ^###^p<0.001 vs CSE group).

## Results

### CS exposure induces COPD-like pathological changes and cellular senescence in rat lungs

**Figure 1:**
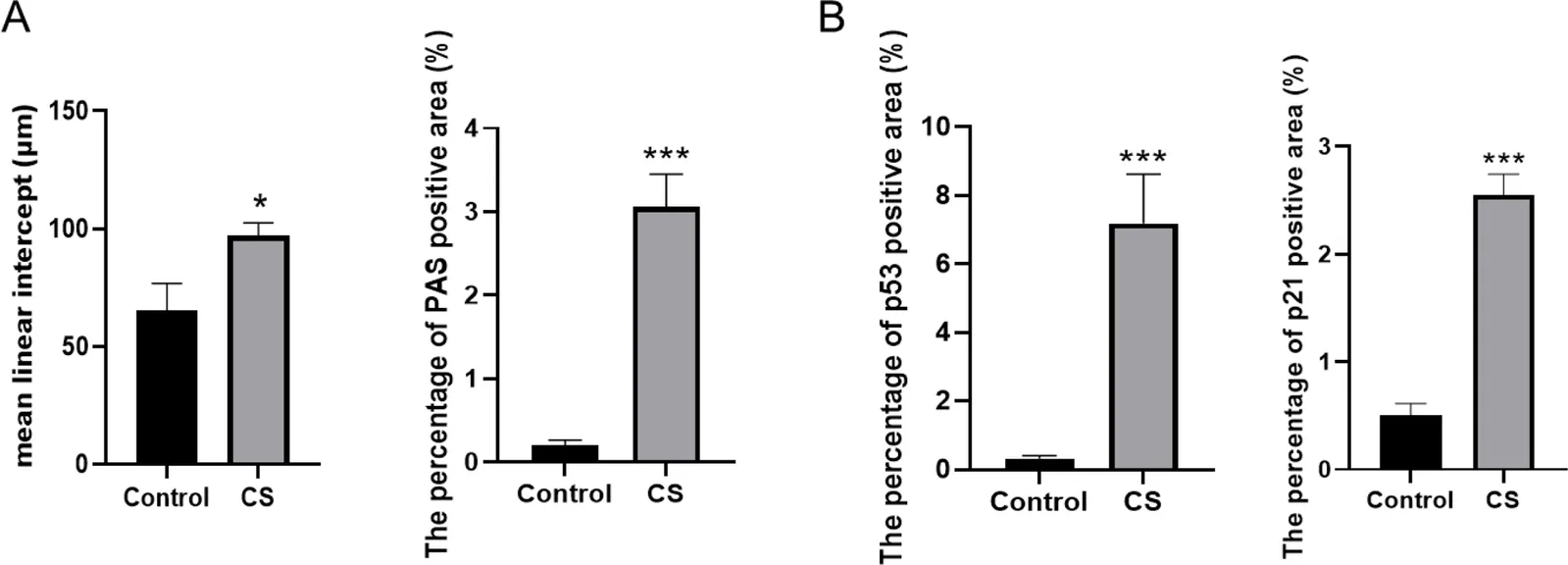
Quantitative summary of cigarette smoke (CS)-induced chronic obstructive pulmonary disease (COPD)-like pulmonary remodeling and epithelial cellular senescence in rat lungs: A) Bar graphs quantify mean linear intercept (alveolar enlargement index), percentage of PAS-positive goblet cells; B) fractional p53/p21 immunohistochemistry (IHC) staining area, and normalized fold changes of p53/p21 protein expression in control rats vs rats subjected to 8 weeks of whole-body CS exposure

Eight weeks of whole-body cigarette smoke (CS) inhalation was performed to establish a rat chronic obstructive pulmonary disease (COPD) model. Quantitative morphometric analysis confirmed hallmark emphysematous remodeling in CS-challenged rat lungs: the mean linear intercept (MLI) was markedly elevated to 112.4 µm (95% CI: 105.7–119.1) versus 68.2 µm (95% CI: 64.3–72.1) in air-exposed control rats, consistent with alveolar dilation and thinning of alveolar septa (Figure 1). Periodic Acid-Schiff (PAS) quantification further demonstrated severe airway mucus hypersecretion in CS animals; the proportion of PAS-positive goblet cells reached 3.12% (95% CI: 2.78–3.46), far exceeding the baseline 0.37% (95% CI: 0.29–0.45) measured in controls (Figure 1). Representative hematoxylin-eosin (HE) and PAS histology micrographs visualizing these structural and secretory changes are provided in Supplementary file Figure S1.

There were n=6 rats per group for histomorphometry and IHC; and n=3 independent biological replicates for western blot quantification. All representative histological micrographs, IHC images and raw western blot bands supporting these aggregate quantitative data are available in Supplementary file Figure S1. Data are presented as mean ± SEM. *p<0.05, ***p<0.001 compared with the control group.

We subsequently quantified senescence biomarker abundance in rat pulmonary tissue. Immunohistochemistry (IHC) scoring revealed drastically expanded p53- and p21-positive epithelial areas within small airways and alveoli of CS-treated rats (Figure 1). The fractional p53 staining area was 7.05% (95% CI: 6.43–7.67) in the CS group compared with 0.65% (95% CI: 0.41–0.89) in controls, while p21-positive coverage increased from 0.28% (95% CI: 0.17–0.39) to 2.53% (95% CI: 2.24–2.82). Western blot densitometry validated these IHC findings at the protein level: CS exposure induced a 2.46-fold upregulation of p53 (95% CI: 2.11–2.81) and a 2.28-fold elevation of p21 (95% CI: 1.97–2.59) relative to control lung lysates (Figure 1). All original IHC micrographs, raw western blot bands and corresponding full quantitative panels supporting these summary bar charts are given in Supplementary file Figure S1 for high-resolution viewing.

### CSE dose-dependently induces cellular senescence in BEAS-2B cells

**Figure 2:**
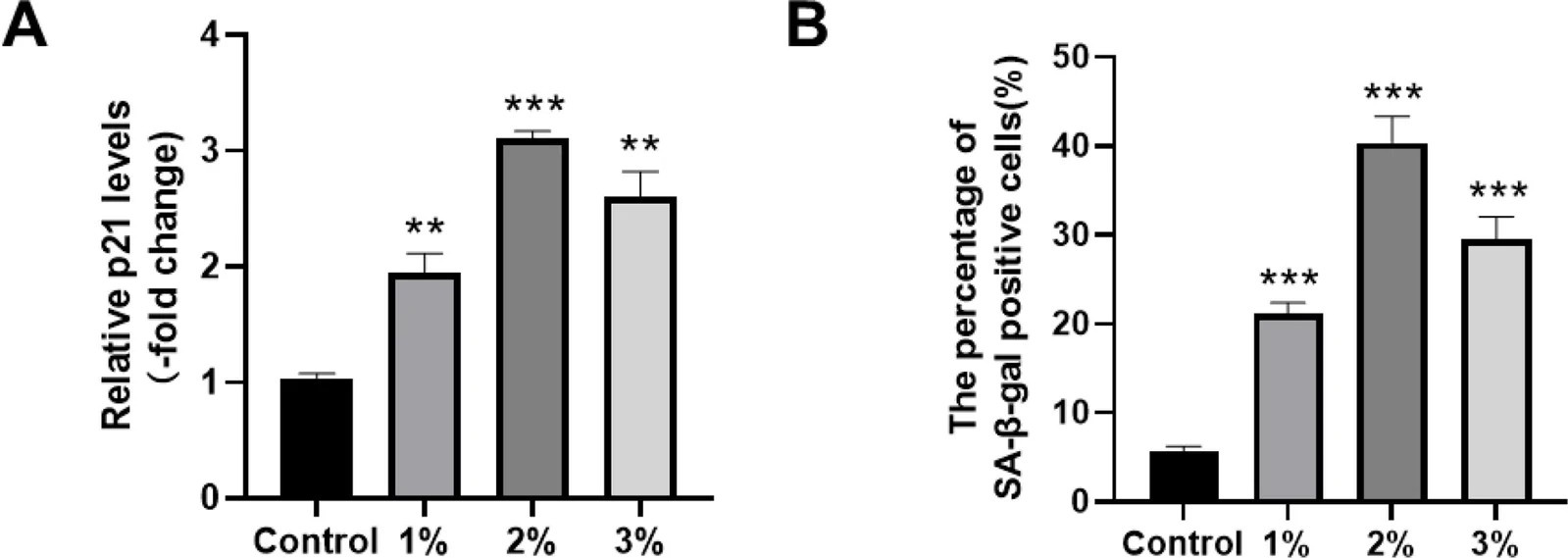
Dose-dependent induction of cellular senescence by cigarette smoke extract (CSE) in BEAS-2B human bronchial epithelial cells. Bar charts summarize normalized p21 protein expression levels (A) and the percentage of senescence-associated β-galactosidase (SA-β-gal)-positive senescent cells (B) after 24 h incubation with 0% (control), 1%, 2% or 3% CSE

Human bronchial epithelial BEAS-2B cells were exposed to serial concentrations (1%, 2%, 3%) of cigarette smoke extract (CSE) for 24 hours to evaluate dose-dependent senescence induction. Quantitative densitometry of western blot bands confirmed dose-dependent elevation of senescence marker p21 protein (Figure 2). Relative p21 expression normalized to GAPDH was 1.00 (95% CI: 0.87–1.13) in untreated control cells, rising to 1.91 (95% CI: 1.66–2.16) with 1% CSE, peaking at 3.06 (95% CI: 2.72–3.40) under 2% CSE, and declining slightly to 2.39 (95% CI: 2.08–2.70) following 3% CSE treatment. Consistent with p21 protein changes, quantitative scoring of senescence-associated β-galactosidase (SA-β-gal) staining revealed a dose-dependent rise in senescent cell proportion, with the maximal effect observed at the 2% CSE dose (Figure 2). Specifically, SA-β-gal-positive senescent cells accounted for 41.7% (95% CI: 38.5–44.9) of the total cell population after 2% CSE stimulation, compared with a baseline of merely 6.3% (95% CI: 4.8–7.8) in vehicle-treated controls. Representative raw western blot images and micrographs of SA-β-gal-stained cells for all CSE concentration groups are provided in Supplementary file Figure S2 in high resolution. On the basis of these dose–response findings, 2% CSE was adopted as the standard stimulation condition for all subsequent *in vitro* functional assays

There were n=3 independent biological replicates. Representative raw western blot strips and SA-β-gal staining micrographs for each treatment group are available in Supplementary file Figure S2. Data are presented as mean ± SEM. **p<0.01, ***p<0.001 vs control group.

### CSE induces cellular senescence via autophagic flux impairment

**Figure 3:**
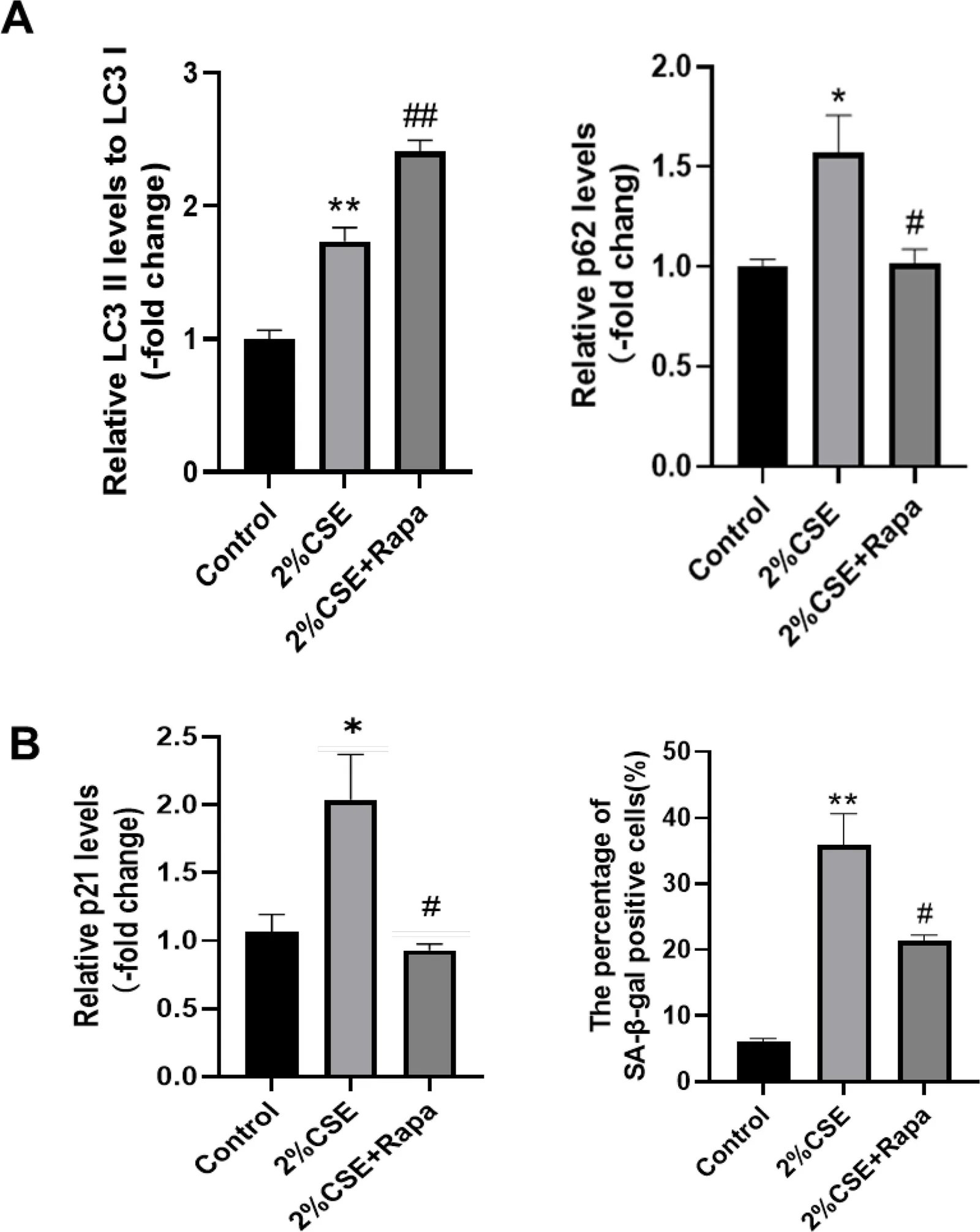
Rapamycin-mediated autophagy restoration reverses CSE-induced autophagic flux dysfunction and cellular senescence in BEAS-2B cells: A) Bar charts showing quantitative densitometry of LC3-II/I ratio and normalized p62 protein expression across control, 2% CSE, and 2% CSE + rapamycin groups; B) Bar charts summarizing relative p21 protein abundance, p21 immunofluorescence mean fluorescence intensity (MFI), and percentage of SA-β-gal-positive senescent cells in the three treatment groups

We next quantified autophagic flux biomarkers in BEAS-2B cells stimulated with 2% CSE. Western blot densitometry revealed disrupted autophagic turnover upon CSE exposure: the LC3-II/I ratio (indicator of autophagosome formation) was elevated, alongside marked accumulation of p62, a canonical marker of blocked autophagic degradation (Figure 3A). In vehicle control cells, the LC3-II/I ratio was 1.02 (95% CI: 0.89–1.15) and normalized p62 expression was set as 1.00 (95% CI: 0.86–1.14). Following 2% CSE stimulation, the LC3-II/I ratio increased to 1.71 (95% CI: 1.53–1.89), and p62 protein abundance rose to 1.62-fold relative to controls (95% CI: 1.44–1.80).

There were n=3 independent replicates. All raw western blot bands, immunofluorescence micrographs and SA-β-gal staining images are available in Supplementary file Figure S3 for high-resolution viewing. Data are presented as mean ± SEM. *p<0.05, **p<0.01 vs control group; ^#^p<0.05, ^##^p<0.01 vs 2% CSE group.

To verify whether restoring autophagy could rescue CSE-mediated senescence, we co-administered the autophagy inducer rapamycin with 2% CSE. Quantitative analysis confirmed rapamycin effectively rescued CSE-impaired autophagic flux (Figure 3A). In cells co-treated with CSE and rapamycin, the LC3-II/I ratio further increased to 2.32 (95% CI: 2.11–2.53), while p62 levels declined to 1.15 (95% CI: 0.99–1.31), nearly matching the baseline control value of 1.00 (95% CI: 0.86–1.14).

Rapamycin also robustly mitigated CSE-triggered cellular senescence signatures (Figure 3B). Western blot quantification demonstrated that rapamycin reversed CSE-induced p21 overexpression: normalized p21 levels reached 2.01 (95% CI: 1.78–2.24) in the CSE-only group, but fell to 0.85 (95% CI: 0.73–0.97) upon rapamycin co-treatment, approximating control levels (1.00; 95% CI: 0.87–1.13). Immunofluorescence quantification of p21 mean fluorescence intensity (MFI) and SA-β-gal senescent cell counting further validated this protective effect. The p21 MFI was 62.80 (95% CI: 59.75–65.85) in CSE-exposed cells and dropped to 24.30 (95% CI: 21.56–27.04) after rapamycin intervention (control baseline =18.20; 95% CI: 15.93–20.47). Consistently, the fraction of SA-β-gal-positive senescent cells decreased from 42.12% (95% CI: 39.05–45.19) under CSE alone to 21.87% (95% CI: 19.24–24.50) with rapamycin co-treatment, close to the control proportion of 7.96% (95% CI: 6.31–9.61).

All original western blot strips, p21 immunofluorescence confocal micrographs, and SA-β-gal bright-field staining images for control, 2% CSE and 2% CSE+ rapamycin groups are provided in high-resolution format within Supplementary file Figure S3.

### CSE-induced ROS accumulation mediates autophagic flux impairment and cellular senescence

We next assessed whether the ROS scavenger N-acetylcysteine (NAC) could reverse CSE-triggered autophagic dysfunction and cellular senescence in BEAS-2B cells. Cells were divided into three experimental groups: vehicle control, 2% CSE alone, and 2% CSE co-incubated with NAC, followed by parallel measurements of intracellular ROS, autophagy markers and senescence readouts.

**Figure 4:**
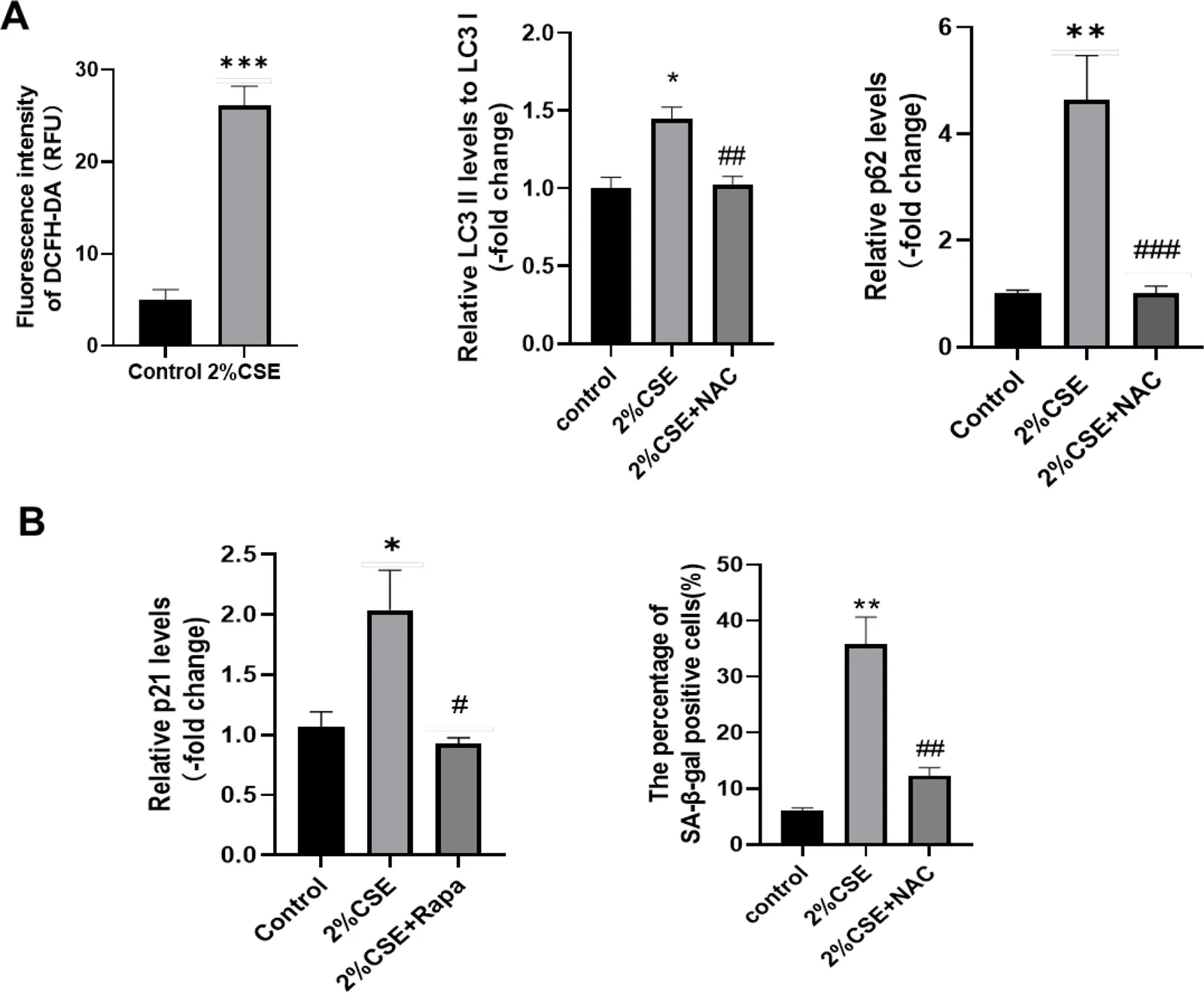
ROS scavenging by NAC restores autophagic flux and ameliorates CSE-induced cellular senescence in BEAS-2B bronchial epithelial cells; A) Bar charts quantifying relative DCFH-DA ROS fluorescence intensity, LC3-II/I ratio, and normalized p62 protein expression in control, 2% CSE, and 2% CSE + NAC groups; B) Bar charts summarizing normalized p21 protein levels, and percentage of SA-β-gal-positive senescent cells across the three treatment groups

Quantitative DCFH-DA fluorescence analysis revealed massive ROS overproduction upon CSE stimulation (Figure 4A). Relative ROS fluorescence intensity stood at 5.23 (95% CI: 4.71–5.75) in control cells, and sharply rose to 25.82 (95% CI: 23.41–28.23) after exposure to 2% CSE

There were n=3 independent replicates. All original imaging data (ROS fluorescence, western blots, immunofluorescence, SA-β-gal staining micrographs) are ginen in Supplementary file Figure S4 for high-resolution viewing. Data are expressed as mean ± SEM. *p<0.05, **p<0.01, ***p<0.001 vs control group; ^#^p<0.05, ^##^p<0.01, ^###^p<0.001 vs 2% CSE group.

Western blot quantification of autophagy biomarkers demonstrated that ROS elimination by NAC rescued impaired autophagic flux (Figure 4A). In cells treated solely with 2% CSE, normalized p62 protein expression increased to 4.08 (95% CI: 3.72–4.44), whereas co-administration of NAC reduced p62 levels to 1.12 (95% CI: 0.94–1.30), nearly returning to the control baseline of 1.00 (95% CI: 0.83–1.17). Meanwhile, the LC3-II/I ratio was 1.84 (95% CI: 1.61–2.07) under CSE alone, and further elevated to 2.26 (95% CI: 2.03–2.49) in the CSE + NAC group, indicating restored autophagosome turnover relative to CSE-only cells (control LC3-II/I baseline =1.00; 95% CI: 0.86–1.14).

Consistent with restored autophagy, NAC intervention robustly alleviated CSE-induced senescence phenotypes (Figure 4B). Densitometry of western blots showed that NAC abrogated p21 overexpression: relative p21 abundance was 2.04 (95% CI: 1.81–2.27) in CSE-exposed cells, and fell to 1.06 (95% CI: 0.92–1.20) with NAC co-treatment, comparable to control levels (1.00, 95% CI: 0.85–1.15). Quantification of p21 immunofluorescence mean fluorescence intensity (MFI) supported this result: p21 MFI reached 63.00 (95% CI: 60.12–65.88) in the CSE group and dropped to 18.50 (95% CI: 16.21–20.79) after NAC treatment (control baseline =24.60; 95% CI: 22.03–27.17). SA-β-gal staining counting further confirmed the anti-senescent effect of NAC: the proportion of SA-β-gal-positive senescent cells was 34.30% (95% CI: 31.52–37.08) with CSE alone, and declined to 12.80% (95% CI: 10.64–14.96) upon NAC co-administration, approaching the control baseline of 6.70% (95% CI: 5.13–8.27).

All raw DCFH-DA ROS fluorescence micrographs, western blot bands for autophagy and senescence proteins, p21 immunofluorescence confocal images, and SA-β-gal staining bright-field photographs across all three treatment groups are provided in high-resolution downloadable format in Supplementary file Figure S4.

### AhR Overactivation mediates CSE-induced ROS accumulation, autophagic dysfunction and cellular senescence

Transcriptome profiling was performed on lung tissues from control and 8-week CS-exposed rats, followed by integrative reanalysis of three public human COPD transcriptomic datasets deposited in the NCBI GEO repository (GSE10006, GSE11784, GSE20257). Overlap analysis via Venn diagram identified 10 shared differentially expressed genes (DEGs) between the rat smoking model and clinical human COPD cohorts. Heatmap and volcano plot visualization highlighted robust upregulation of canonical AhR downstream target genes CYP1A1 and CYP1B1 in both CS-injured rat lung tissue and patient COPD specimens. Aggregated quantification of GEO datasets further validated that CYP1A1 and CYP1B1 transcript abundance was significantly higher in smokers than non-smokers, with maximal expression detected in smokers diagnosed with COPD (Supplementary file Figure S5A).

We next cross-validated AhR signaling hyperactivation at the transcriptional level in both in vivo rat lungs and in vitro BEAS-2B bronchial epithelial cells using qRT-PCR quantification (Supplementary file Figure S5A). In rat pulmonary tissue, CYP1A1 mRNA was dramatically induced by CS exposure: relative expression reached 24.30 (95% CI: 22.04–26.56) in CS-challenged rats versus the control baseline of 1.00 (95% CI: 0.85–1.15). Similarly, CYP1B1 transcript levels increased from 1.00 (95% CI: 0.82–1.18) in controls to 5.85 (95% CI: 4.03–7.57) following chronic CS inhalation. In cultured BEAS-2B cells, 2% CSE stimulation also markedly elevated AhR target transcripts: Cyp1a1 expression rose from 1.00 (95% CI: 0.84–1.16) in untreated cells to 4.15 (95% CI: 3.68–4.62) under CSE, while Cyp1b1 increased from 0.87 (95% CI: 0.74–1.00) to 1.36 (95% CI: 1.19–1.53).

To establish the causal role of AhR hyperactivation upstream of CSE-mediated lung injury, BEAS-2B cells were pre-incubated with the selective AhR antagonist CH223191 prior to 2% CSE stimulation. qRT-PCR quantification confirmed that AhR blockade fully reversed CSE-triggered induction of Cyp1a1 and Cyp1b1 (Supplementary file Figure S5B). For Cyp1a1, transcript levels peaked at 3.67 (95% CI: 3.22–4.12) with CSE alone and fell back to 0.87 (95% CI: 0.74–1.00) after CH223191 co-treatment; Cyp1b1 expression shifted from 1.36 (95% CI: 1.19–1.53) in CSE-only cultures to 0.14 (95% CI: 0.11–0.17) upon AhR inhibition.

DCFH-DA quantitative fluorescence measurements demonstrated that pharmacological AhR suppression alleviated CSE-dependent intracellular ROS overproduction (Supplementary file Figure S5B). Baseline ROS fluorescence intensity was 5.23 (95% CI: 4.71–5.75) in control cells, surged to 25.82 (95% CI: 23.41–28.23) under CSE, and was partially rescued to 14.92 (95% CI: 13.05–16.79) in cells co-treated with CSE and CH223191. Complementary qRT-PCR analysis of oxidative stress signature genes (Sod2, Nrf2, Hmox1, Nqo1) further confirmed AhR inhibition restored homeostatic antioxidant transcriptional profiles disrupted by CSE (Supplementary file Figure S5B). CSE exposure suppressed Sod2 expression to 0.62 (95% CI: 0.55–0.69), which recovered to 1.10 (95% CI: 0.87–1.25) with CH223191. Conversely, CSE induced marked upregulation of antioxidant response genes: Nrf2 (1.92; 95% CI: 1.81–2.24), Hmox1 (2.03; 95% CI: 1.95–2.36), and Nqo1 (3.54; 95% CI: 3.29–3.75); and AhR antagonism reduced these values to 1.32, 1.14 and 1.55 respectively.

Western blot densitometry of autophagy markers revealed that CH223191 normalized CSE-impaired autophagic flux (Supplementary file Figure S5C). CSE alone drove robust p62 protein accumulation (1.90; 95% CI: 1.72–2.15), whereas co-administration of CH223191 lowered p62 abundance to 1.24 (95% CI: 1.10–1.39). The LC3-II/I ratio was moderately elevated to 1.45 (95% CI: 1.28–1.56) after CSE treatment and further increased to 2.02 (95% CI: 1.83–2.39) with AhR inhibition, reflecting restored autophagosome clearance capacity.

Finally, quantitative senescence marker readouts verified that AhR antagonism mitigated CSE-induced epithelial senescence (Supplementary file Figure S5C). CSE stimulation increased normalized p21 protein levels to 6.04 (95% CI: 5.73–6.55), which decreased to 3.73 (95% CI: 3.48–4.06) upon CH223191 co-treatment. Quantification of p21 immunofluorescence mean fluorescence intensity mirrored this trend: values shifted from 66.02 (95% CI: 61.58–70.25) in CSE-only cells to 15.83 (95% CI: 13.25–18.04) with AhR blockade. Consistent with molecular data, SA-β-gal senescent cell counts declined from 35.85% (95% CI: 30.22–41.28) under CSE alone to 19.00% (95% CI: 16.61–21.05) following CH223191 intervention.

All raw multi-omics visualizations (Venn diagram, heatmap, volcano plot), original qPCR bar charts for clinical GEO cohorts, DCFH-DA ROS micrographs, western blot bands for CYP proteins and autophagy/senescence markers, p21 immunofluorescence confocal images, and SA-β-gal bright-field staining micrographs are provided as high-resolution downloadable files within Supplementary file Figure S6.

## Discussion

We identified a novel AhR-ROS-autophagy axis mediating CS-induced pulmonary cellular senescence, a key pathogenic mechanism in COPD. Our main findings are: 1) CS exposure induces COPD-like pathological changes and cellular senescence in rat lungs, and CSE dose-dependently induces senescence in human bronchial epithelial cells; 2) CSE induces cellular senescence via autophagic flux impairment, and restoration of autophagy by rapamycin alleviates senescence; 3) CSE-induced ROS accumulation mediates autophagic flux impairment, and ROS scavenging by NAC reverses autophagy dysfunction and senescence; 4) CS/CSE activates the AhR signaling pathway in pulmonary tissues/cells; and 5) Blockade of AhR by CH223191 reduces CSE-induced ROS production, restores autophagic flux, and ameliorates cellular senescence. These findings reveal a new molecular cascade linking CS exposure to pulmonary cellular senescence, and identify the AhR-ROS-autophagy axis as a potential therapeutic target for COPD.

Cellular senescence is a critical driver of COPD pathogenesis, and targeting senescence has emerged as a promising therapeutic strategy^5,8^. Our study confirmed that CS exposure induces significant cellular senescence in rat lungs and CSE induces dose-dependent senescence in BEAS-2B cells, consistent with previous studies^7,9^. We further found that autophagic flux impairment is a key mediator of CSE-induced senescence. CS-induced autophagy dysfunction is characterized by increased autophagy initiation but impaired degradation, leading to p62 accumulation^11,24^. Our results showed that CSE increased the LC3-II/I ratio and p62 expression, and rapamycin (which restores autophagic flux) significantly attenuated senescence. This is consistent with a previous study showing that rapamycin ameliorates CS-induced lung injury and senescence^15^, confirming that autophagy activation is a potential strategy to inhibit CS-induced lung aging.

Oxidative stress is a well-recognized link between CS exposure and autophagy dysfunction^14,25^. Our study showed that CSE induces excessive ROS production in BEAS-2B cells, and NAC (ROS scavenger) not only reduces ROS levels but also restores autophagic flux and alleviates senescence. This is consistent with previous studies showing that oxidative stress impairs autophagic flux by inhibiting lysosomal function^26^, and ROS scavenging reverses autophagy dysfunction in CS-induced lung injury^27^. Our results confirm that ROS is a key intermediate between CS exposure and autophagy impairment, and antioxidant therapy may be an effective strategy to inhibit CS-induced lung cell senescence.

The AhR pathway is a novel upstream regulator of the ROS-autophagy-senescence cascade identified in our study. AhR is highly expressed in lung epithelial cells and is robustly activated by CS components^16,17^. Excessive AhR activation induces the expression of CYP1A1 and CYP1B1, and inefficient catalytic cycling of these enzymes leads to increased ROS production^20^. Our transcriptomic analysis and GEO dataset mining showed that CYP1A1 and CYP1B1 are significantly upregulated in CS-exposed rat lungs and human COPD samples, and their expression is associated with smoking intensity and COPD progression. We further confirmed that AhR blockade by CH223191 reduces CSE-induced ROS production, restores autophagic flux, and ameliorates senescence. Our findings provide new mechanistic insights into how AhR participates in cigarette-smoke-triggered pulmonary epithelial senescence.

Previous studies have shown that AhR deficiency exacerbates CS-induced pulmonary inflammation and oxidative stress^4,21^, suggesting a protective role of basal AhR activation in acute CS exposure. However, our study showed that excessive AhR activation mediates CS-induced lung cell senescence in chronic CS exposure (8 weeks), indicating a dual role of AhR in CS-induced lung injury: basal AhR activation may be protective against acute inflammation, while excessive/long-term AhR activation leads to oxidative stress, autophagy dysfunction, and senescence in chronic exposure. This dual role may be related to the duration and intensity of CS exposure, and the balance between AhR-mediated detoxification and ROS production. Our findings suggest that inhibition of excessive AhR activation (rather than complete knockout) is a potential therapeutic strategy for chronic CS-induced COPD.

### Strengths and limitations

Our study has several strengths: 1) We combined *in vivo* (rat COPD model), *in vitro* (BEAS-2B cells), and clinical transcriptomic data to validate the AhR-ROS-autophagy axis, enhancing the translational relevance of our findings; 2) We performed multiple rescue experiments (rapamycin, NAC, CH223191) to confirm the causal relationship between each step of the cascade; 3) We used multiple methods to detect each endpoint (eg, senescence was detected by SA-β-gal, WB, IHC, and IF), ensuring the reliability of our results.

However, our study also has some limitations: 1) We only used BEAS-2B cells (immortalized human bronchial epithelial cells) for *in vitro* experiments, and primary human bronchial epithelial cells or patient-derived organoids should be used in future studies to confirm our findings; 2) We only used male rats in the *in vivo* model, and sex differences should be investigated in future studies; 3) We did not perform *in vivo* rescue experiments with CH223191, and future studies should validate the therapeutic potential of AhR inhibition in CS-induced rat COPD models; 4) Although we performed cross-validation with public human COPD transcriptomic datasets obtained from the Gene Expression Omnibus (GEO) repository, the majority of our experimental evidence originates from rat lung tissues and the BEAS-2B human bronchial epithelial cell line. Animal-model and cell-culture findings cannot be fully generalized to real-world human patients. Differences between species, individual heterogeneity among patients, complex clinical co-morbidities and variable smoking exposure patterns may alter the actual role of aryl hydrocarbon receptor (AhR) in human COPD-related epithelial senescence. Further studies using primary human bronchial epithelial cells and clinical patient specimens are needed to confirm the translational relevance of our results.

## Conclusions

Our study reveals a novel molecular cascade wherein CS exposure activates AhR, leading to excessive ROS production, autophagic flux impairment, and ultimately pulmonary cellular senescence (Supplementary file Figure S7). The AhR-ROS-autophagy axis is a new pathogenic pathway mediating CS-induced lung aging, and targeting this axis (eg, AhR antagonists, ROS scavengers, autophagy inducers) may represent a promising therapeutic strategy for COPD. Our findings provide pre-clinical experimental evidence highlighting the potential biological relevance of AhR for COPD-associated epithelial senescence.

## Abbreviations

AhR: aryl hydrocarbon receptor
COPD: chronic obstructive pulmonary disease
CS: cigarette smoke
CSE: cigarette smoke extract
ROS: reactive oxygen species
NAC: N-acetylcysteine
WB: Western blotting
IHC: immunohistochemistry
IF: immunofluorescence
SA-β-gal: senescence-associated β-galactosidase
qRT-PCR: quantitative real-time PCR
DEGs: differentially expressed genes
GEO: Gene Expression Omnibus
BALF: bronchoalveolar lavage fluid
HE: hematoxylin-eosin
PAS: Periodic Acid-Schiff
DCFH-DA: 2’,7’-dichlorodihydrofluorescein diacetate
MFI: mean fluorescence intensity
IOD: integrated optical density
