# Supplementary material for: Activation of aryl hydrocarbon receptor by cigarette smoke induces oxidative stress, autophagy impairment and cellular senescence
Source: Tob Induc Dis. 2026 Sep 9;24:10.18332/tid/226746. doi: 10.18332/tid/226746 (PMC13575394; doi:10.18332/tid/226746)
Supplement: Supplementary file 1 [file TID-24-160-s001.pdf]

Tobacco Induced Diseases

**Supplementary file**

© 2026 Hu X.D. et al.

**DOI:**

10.18332/tid/226746

The content has been provided by the author(s) and has not been reviewed, verified, or endorsed by European Publishing. It may not have undergone peer review. The views, opinions, and recommendations expressed are solely those of the author(s) and do not necessarily reflect the position of European Publishing. European Publishing accepts no responsibility or liability for any consequences arising from the use of, or reliance on, this content.

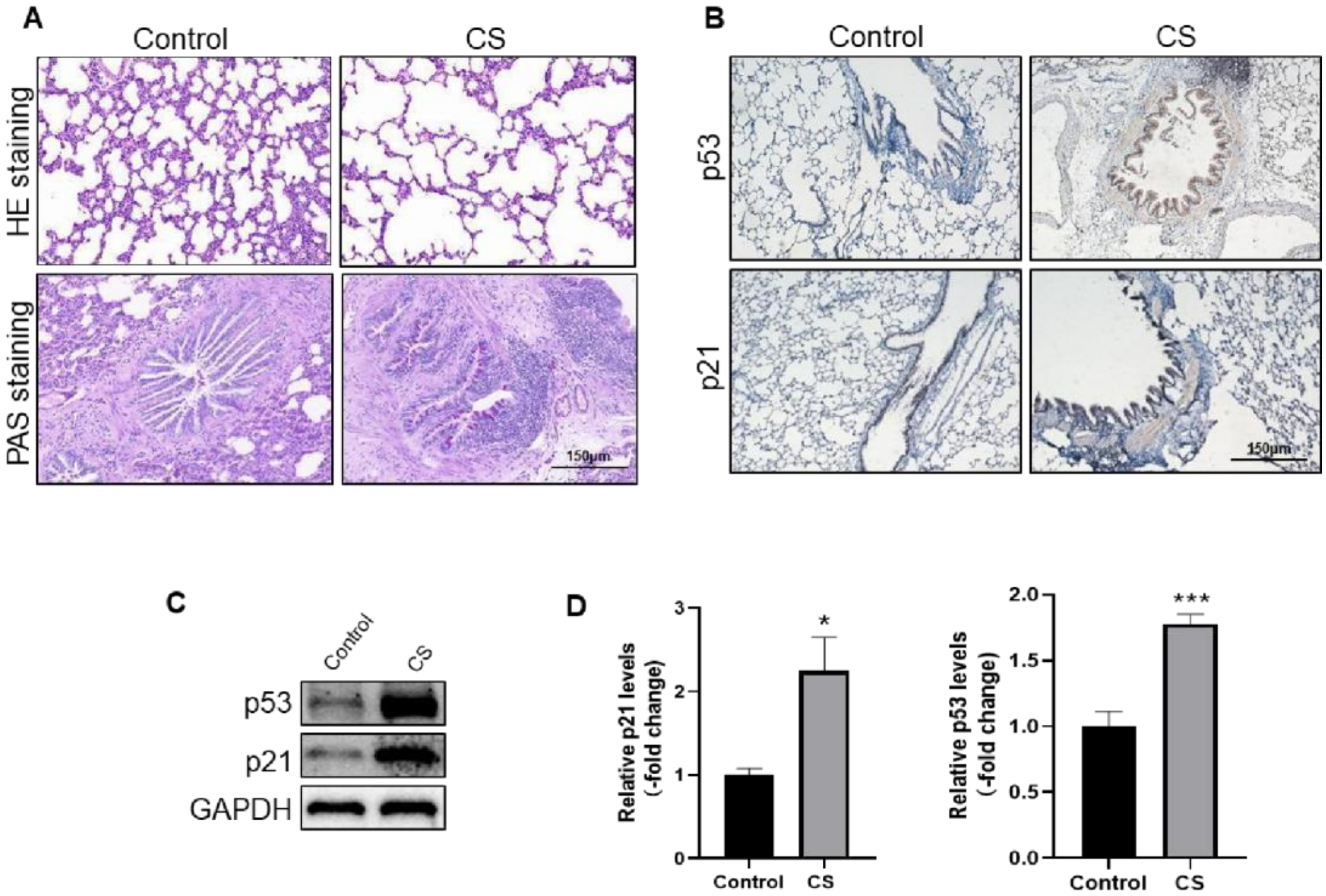


Supplementary Figure S1. Full multi-panel histological, immunohistochemical and western blot characterization of CS-induced lung pathological injury and epithelial senescence in rats, corresponding to the quantitative summary data shown in Main Figure 1.

(A) Representative hematoxylin-eosin (HE) staining (scale bar = 100 μm) and Periodic AcidSchiff (PAS) staining (scale bar = 150 μm) of lung tissues from control and 8-week CSexposed rats. CS exposure triggers obvious alveolar enlargement, thinning of alveolar walls, and prominent airway goblet cell hyperplasia with excessive mucus secretion. (B) Representative IHC staining for senescence markers p53 and p21 in rat lung sections (scale bar = 150 μm). Positive staining is predominantly localized to small airway epithelium and alveolar epithelium in CS-challenged animals.

(C) Original western blot bands showing p53, p21 and GAPDH (loading control) protein expression in lung homogenates of control and CS-exposed rats.

(D) Densitometric quantification of relative p53 and p21 protein levels normalized to GAPDH (n = 3 independent experiments).

All images are provided in high-resolution downloadable format. Data are expressed as mean ± SEM. *P < 0.05, ***P < 0.001 vs. control group.

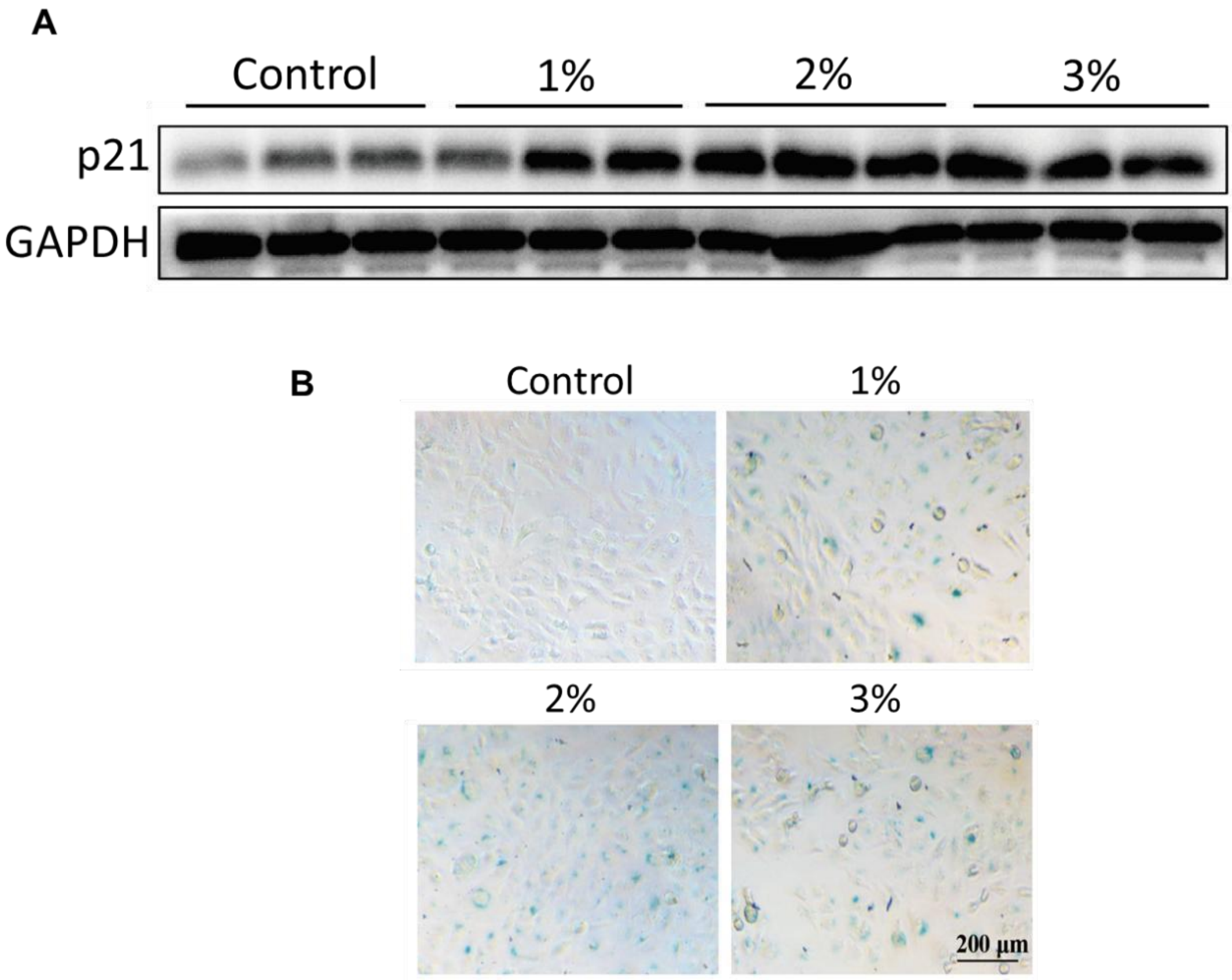


Supplementary Figure S2. Full dose-response characterization of CSE-triggered senescence in BEAS-2B cells, matching quantitative summary data in Main Figure 2. (A) Raw western blot bands illustrating p21 and GAPDH (loading control) protein expression across control, 1%, 2% and 3% CSE groups.

(B) Representative bright-field micrographs of SA-β-gal staining for each CSE concentration group (scale bar = 200 μm); blue cytoplasmic staining marks senescent BEAS-2B cells.

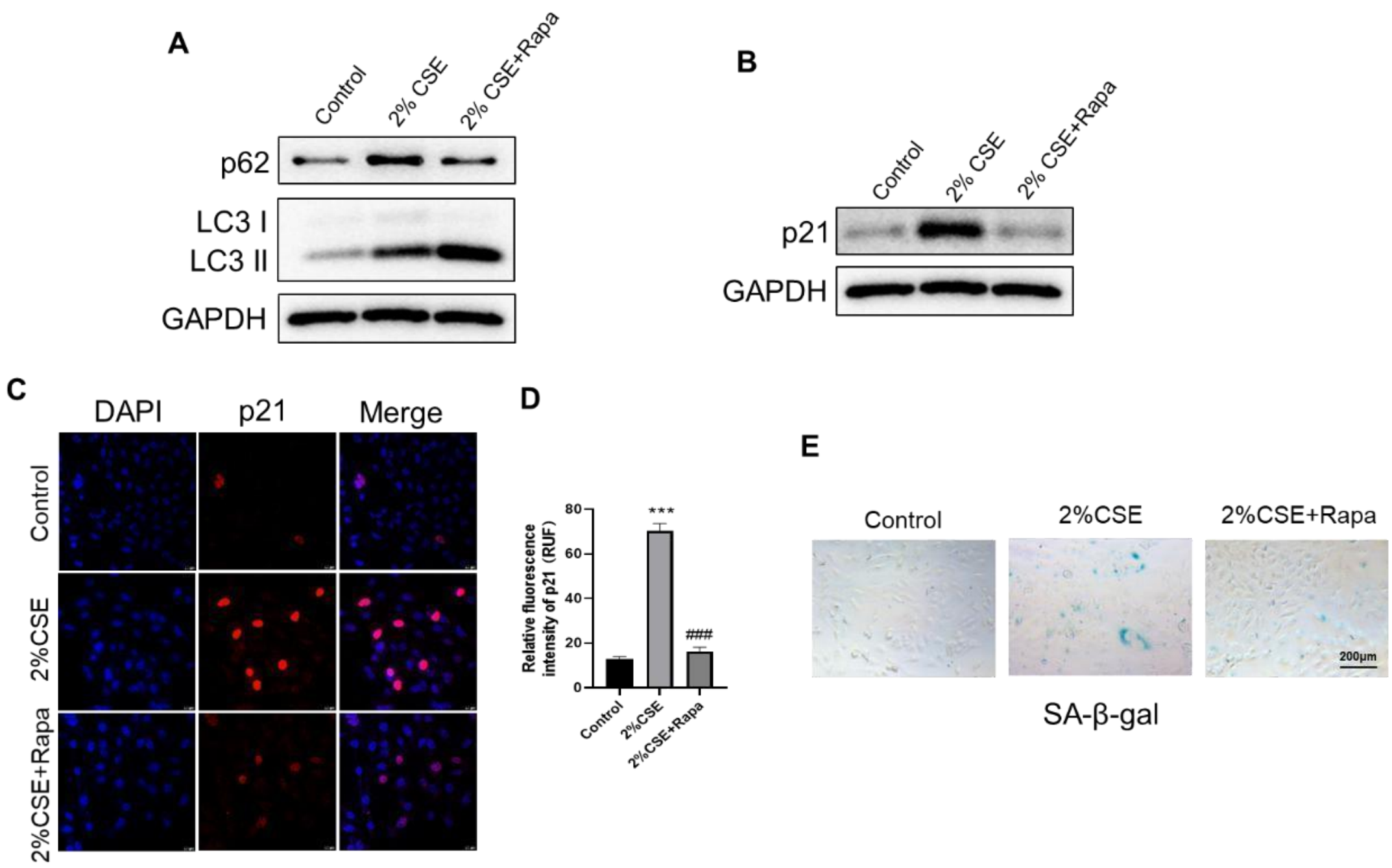


Supplementary Figure S3. Complete experimental imaging and quantification data for rapamycin rescue assays, corresponding to quantitative summary plots in Main Figure 3.

(A) Representative western blot bands detecting LC3-I, LC3-II, p62 and GAPDH loading control in control, 2% CSE and 2% CSE + rapamycin-treated BEAS-2B cells.

(B) Raw western blot bands for senescence marker p21 and GAPDH across all three treatment groups.

(C) Confocal immunofluorescence micrographs of p21 (red) with DAPI nuclear counterstain (blue) (scale bar = 20 μm) for each experimental group. (D) Statistical quantification of p21 mean fluorescence intensity (MFI).

(E) Bright-field SA-β-gal staining micrographs visualizing senescent cells (blue cytoplasm; scale bar = 200 μm).

(H) Bar graph quantifying the percentage of SA-β-gal-positive senescent cells. All panels are supplied in downloadable high-resolution format. Data are expressed as mean ± SEM. ***P < 0.001 vs. control group; ###P < 0.001 vs. 2% CSE group.

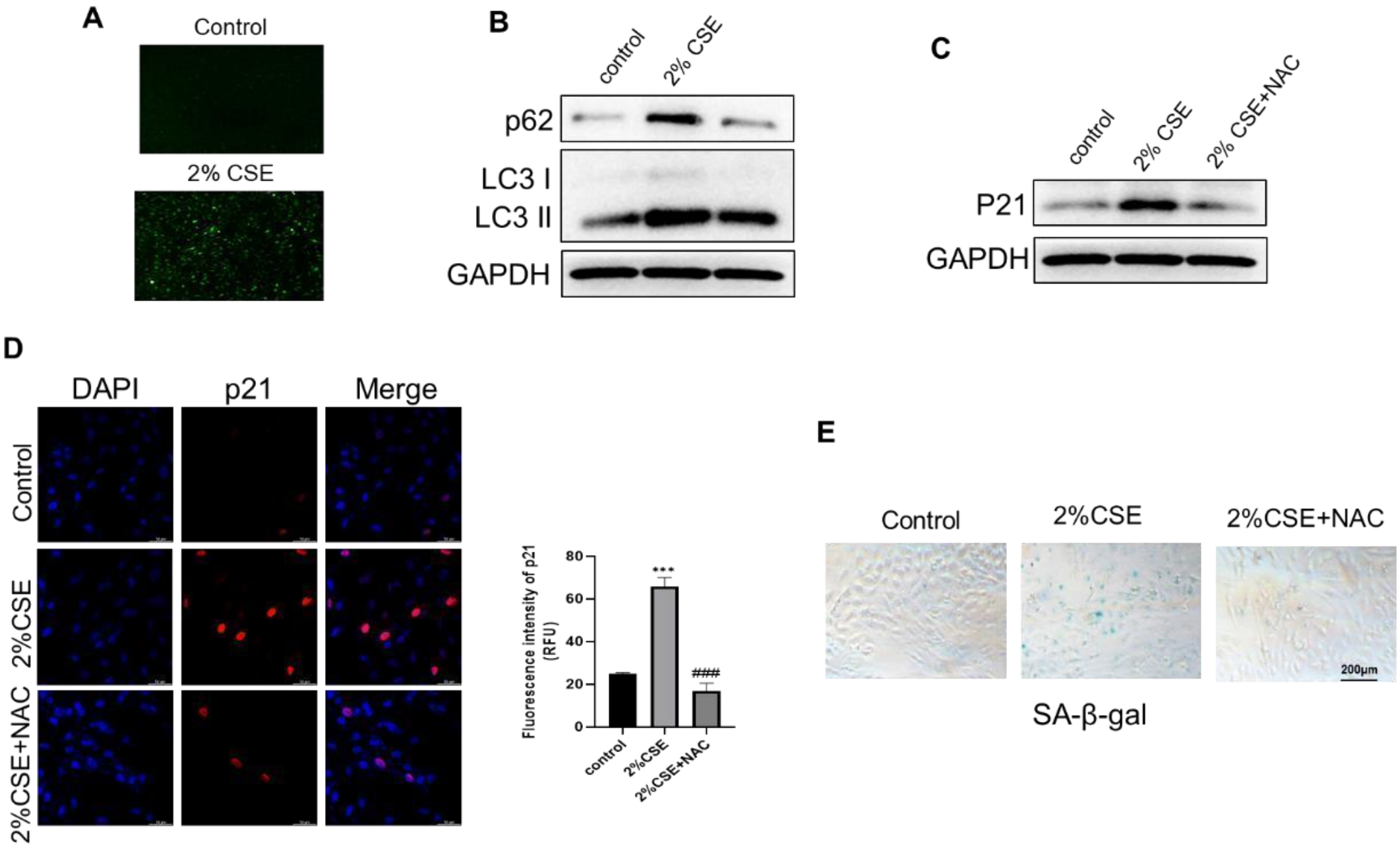


Supplementary Figure S4. Full imaging and quantitative datasets for NAC antioxidant rescue assays, corresponding to the summary bar graphs presented in Main Figure 4. (A) Representative DCFH-DA fluorescence micrographs visualizing intracellular ROS (green signal; scale bar = 200 μm) in control, 2% CSE, alongside quantitative ROS fluorescence intensity analysis.

(B) Raw western blot bands for autophagy markers LC3-I/LC3-II, p62 and GAPDH loading control, with corresponding densitometric quantification of LC3-II/I ratio and relative p62 expression.

(C) Original western blot strips for senescence marker p21 and GAPDH.

(D) Confocal immunofluorescence micrographs of p21 (red) and DAPI nuclear counterstain (blue; scale bar = 20 μm), plus statistical quantification of p21 mean fluorescence intensity (MFI).

(E) Bright-field SA-β-gal staining micrographs showing senescent cells with blue cytoplasmic staining (scale bar = 200 μm), and bar graph quantification of SAβgalpositive cell percentage.

All panels are available for download in high resolution. Data are presented as mean ± SEM. ***P < 0.001 vs. control group; ###P < 0.001 vs. 2% CSE group.

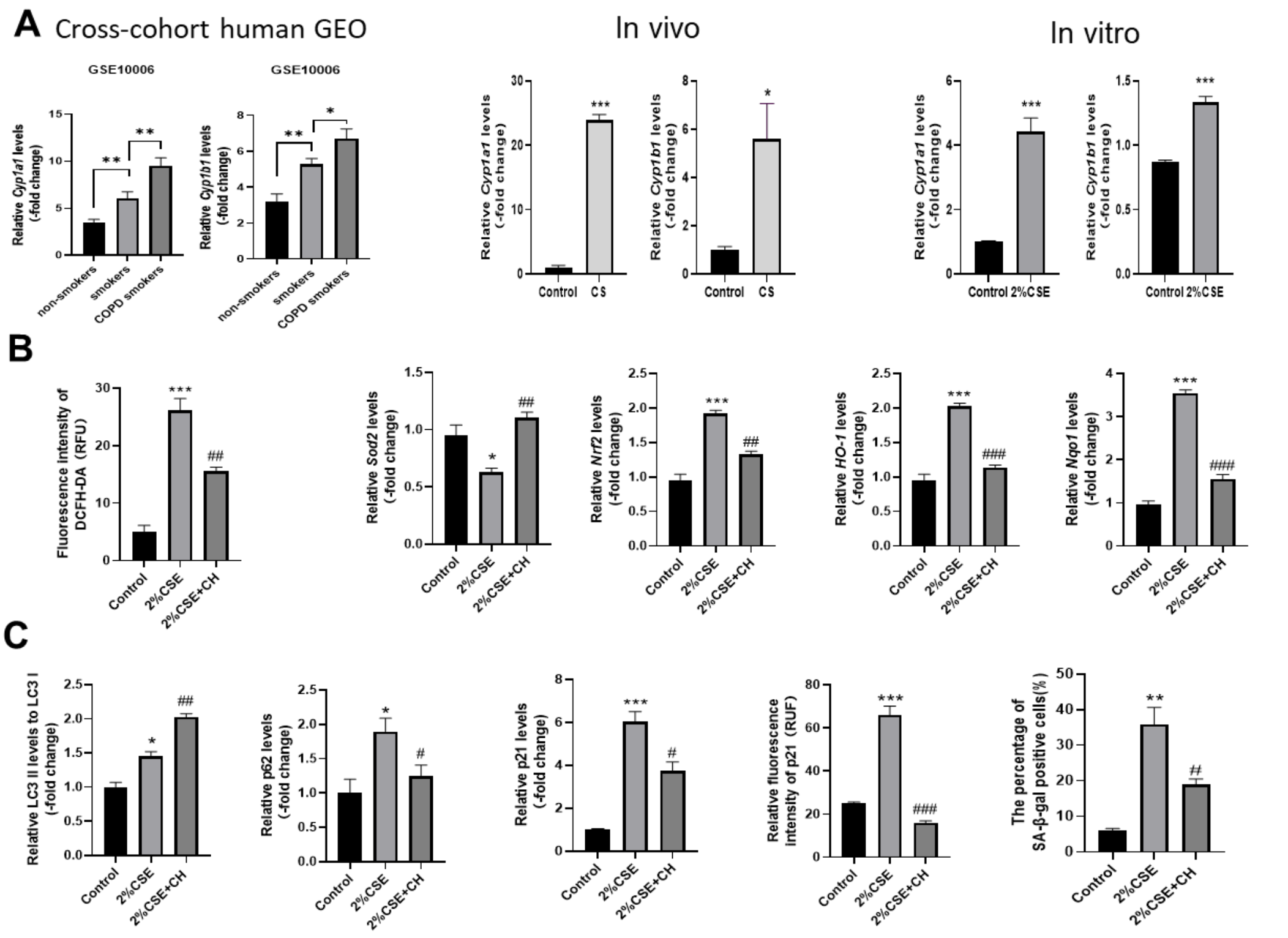


Supplementary Figure S5. Cigarette smoke/CSE drives AhR pathway hyperactivation, and pharmacological AhR inhibition with CH223191 suppresses downstream ROS overproduction, autophagic flux impairment and cellular senescence. (A) Bar chart summary of CYP1A1 and CYP1B1 mRNA expression: cross-cohort human GEO clinical data (non-smokers, healthy smokers, COPD smokers), in vivo rat CS model, and in vitro BEAS-2B CSE stimulation. (B) Quantitative bar graphs for intracellular DCFH-DA ROS fluorescence intensity and relative transcript levels of oxidative stress-related genes (Sod2, Nrf2, Hmox1, Nqo1) across control, 2% CSE, and 2% CSE + CH223191 groups (n = 3 independent replicates). (C) Bar charts quantifying autophagy markers (LC3-II/I ratio, normalized p62), senescence marker p21 protein abundance, p21 immunofluorescence mean fluorescence intensity, and percentage of SA-β-gal-positive senescent cells in the three cell treatment groups (n=3 independent replicates). All multiomics plots (Venn, heatmap, volcano), raw western blot bands, fluorescence staining micrographs and full

uncompressed quantitative panels are deposited in Supplementary Figure S5 for highresolution viewing. Data are presented as mean ± SEM. *p<0.05, **p< 0.01, ***p<0.001 vs. control group; #p<0.05, ##p<0.01, ###p<0.001 vs 2% CSE group.

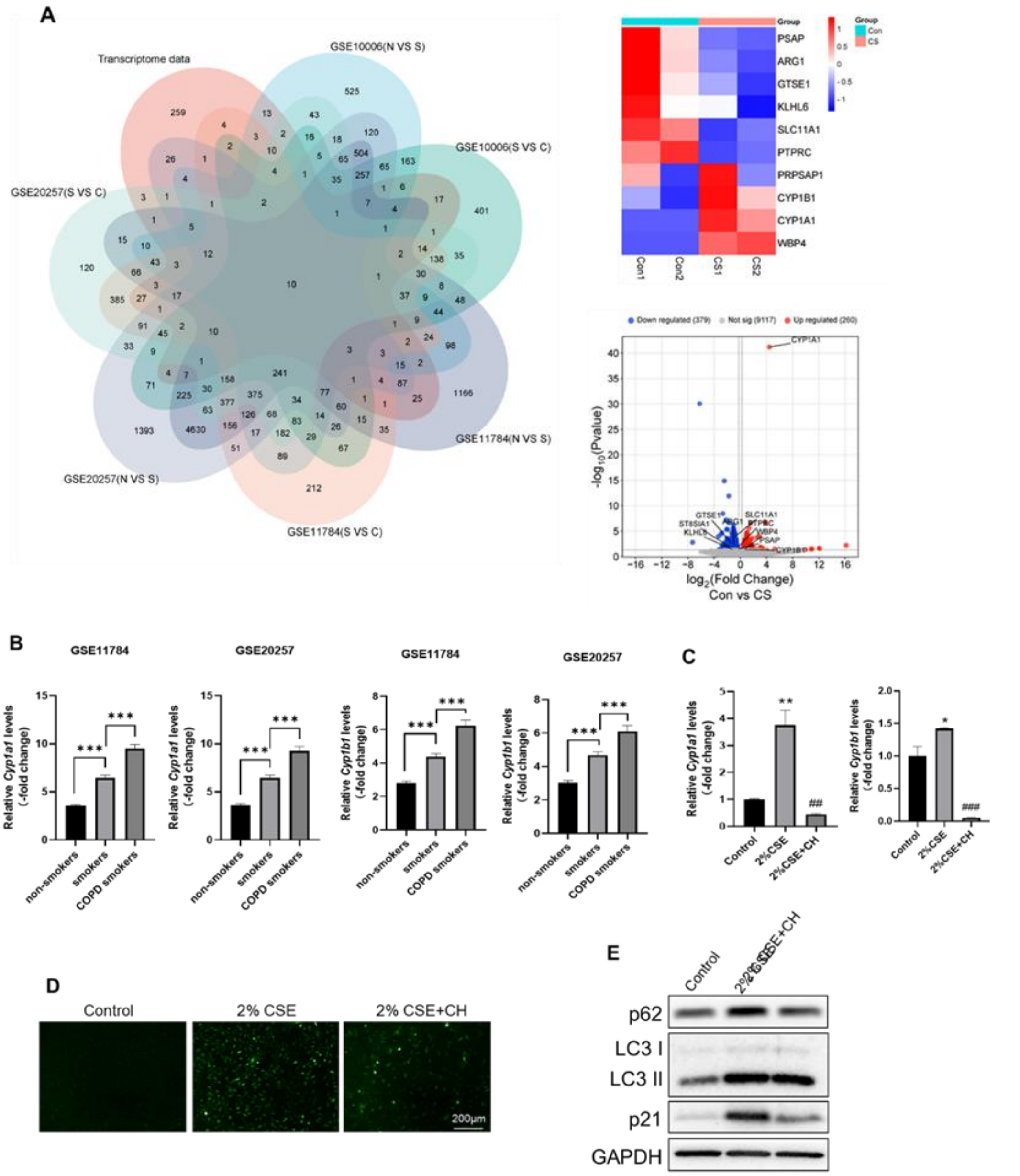

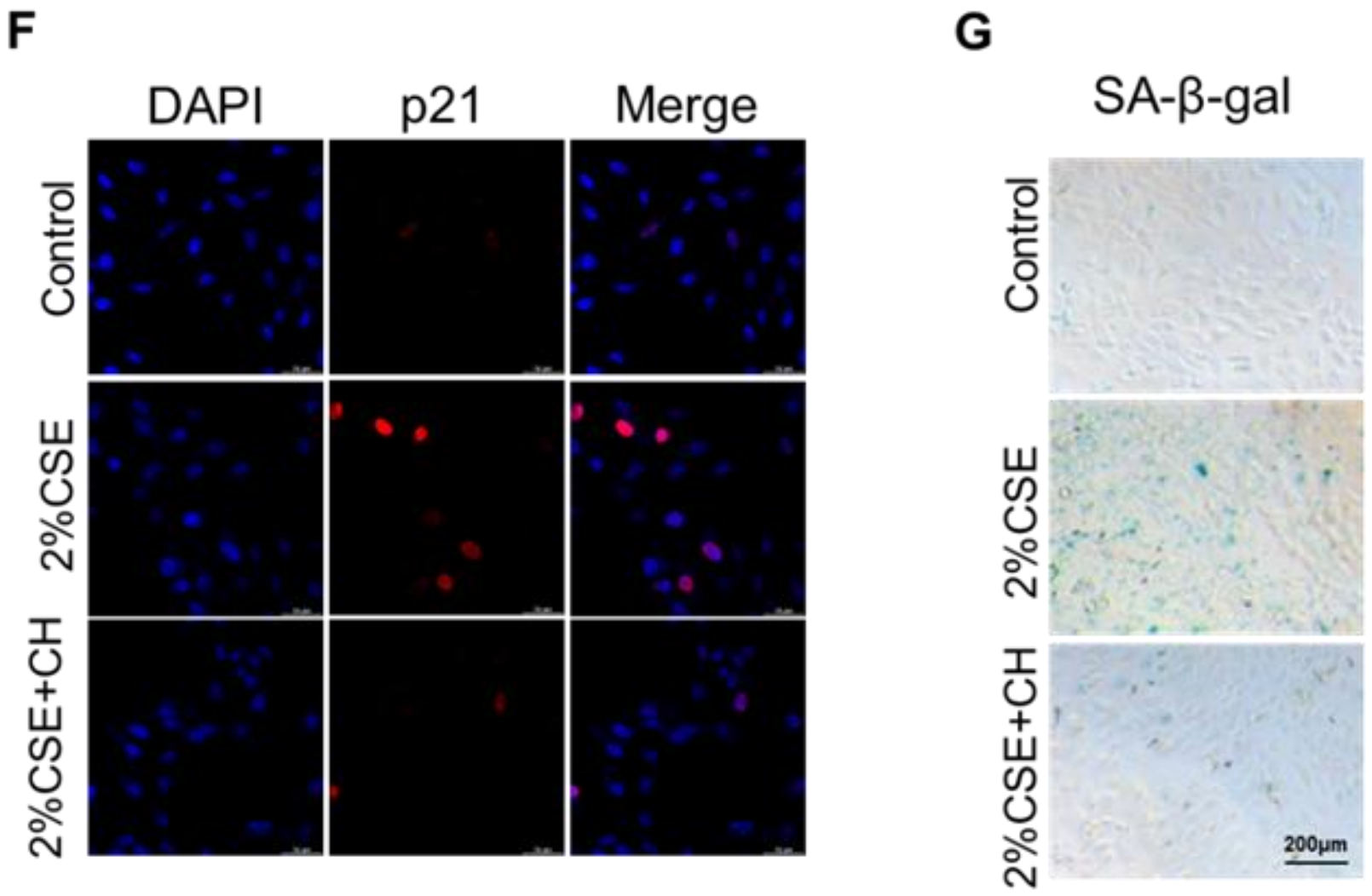


Supplementary Figure S6. Complete multi-omics, molecular and cellular imaging datasets validating AhR as the upstream mediator of CSE-induced ROS-autophagy-senescence cascade, corresponding to summary quantitative plots in Main Figure 5.

(A) Bioinformatic multi-omics panels: Venn diagram of overlapping DEGs between rat CS lung transcriptome and three human COPD GEO datasets; heatmap of shared DEG expression profiles; volcano plot highlighting upregulated CYP1A1/CYP1B1 in CS-exposed rat lung tissue.

(B) Full qPCR quantification of CYP1A1 and CYP1B1 across GEO clinical subgroups (non-smokers, smokers, COPD smokers).

(C) qPCR bar graphs showing Cyp1a1 and Cyp1b1 mRNA expression in BEAS-2B control, 2% CSE, and 2% CSE + CH223191 groups.

(D) Representative DCFH-DA ROS fluorescence micrographs (scale bar = 200 μm).

(E) Raw western blot bands for LC3-I/LC3-II, p62, p21 and GAPDH loading control.

(F) Confocal immunofluorescence micrographs of p21 (red) with DAPI nuclear counterstain (scale bar = 20 μm).

(G) Bright-field SA-β-gal staining micrographs (scale bar = 200 μm) and full quantitative analysis of SA-β-gal-positive senescent cell proportion.

All panels are supplied in high-resolution downloadable format. Data are expressed as mean ± SEM. *$P < 0.05$, **$P < 0.01$, ***$P < 0.001$ vs. control group or non-smokers or smokers; ##$P < 0.01$, ###$P < 0.001$ vs. 2% CSE group.

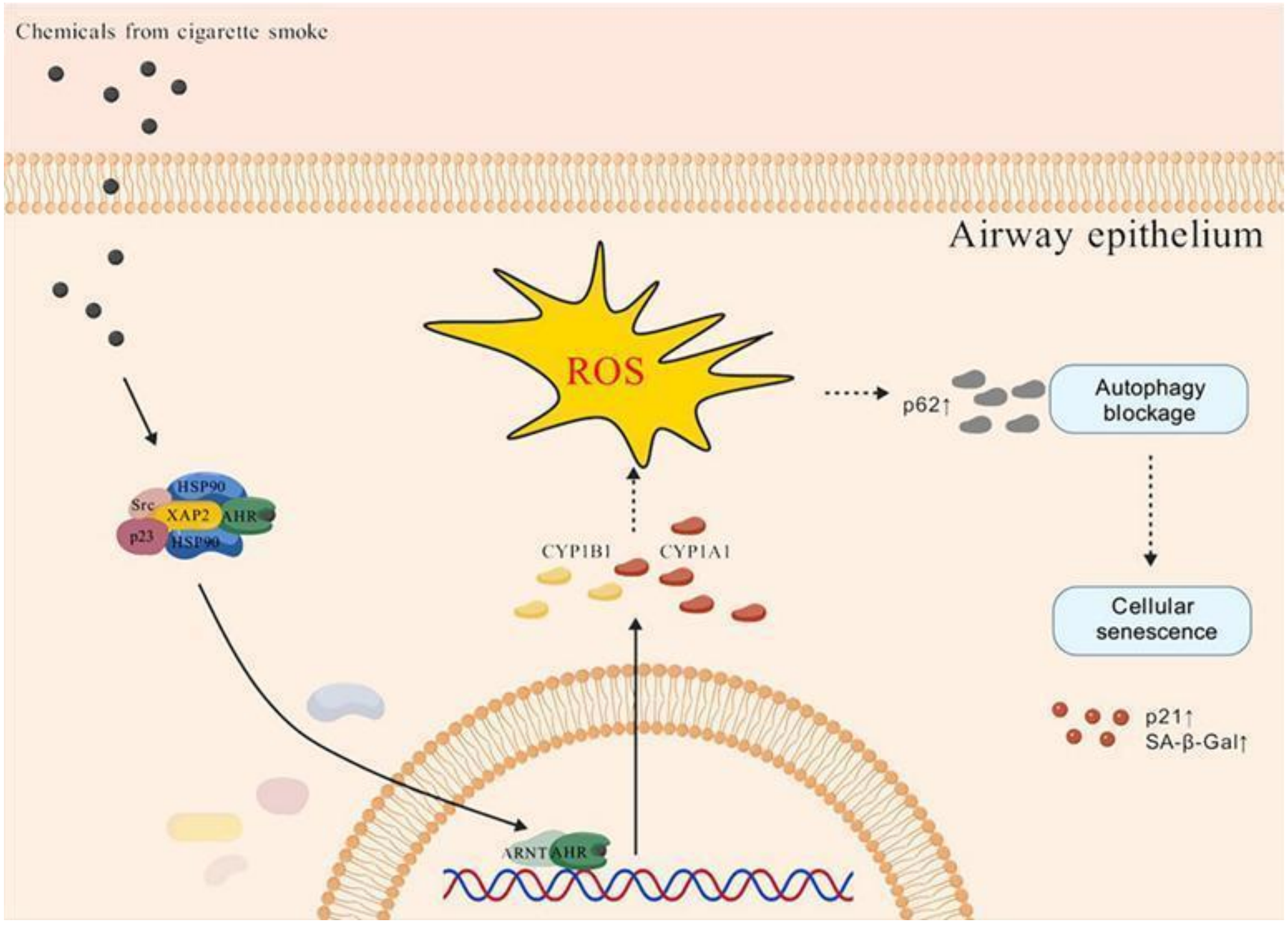


Supplementary Figure S7. Schematic diagram of the AhR-ROS-autophagy axis mediating cigarette smoke (CS)-induced pulmonary cellular senescence in chronic obstructive pulmonary disease (COPD). CS exposure activates the aryl hydrocarbon receptor (AhR) in pulmonary epithelial cells, which upregulates the expression of downstream target genes CYP1A1 and CYP1B1. Excessive AhR activation triggers excessive intracellular

reactive oxygen species (ROS) production and oxidative stress by disrupting CYP catalytic cycling. Elevated ROS levels impair autophagic flux (characterized by increased LC3-II/I ratio and p62 accumulation) by inhibiting lysosomal function and autophagosome degradation. Impaired autophagic flux leads to the accumulation of damaged cellular components, which ultimately induces irreversible cell cycle arrest and cellular senescence (marked by upregulated p53 and p21 expression, increased SA-β-gal-positive cells). Targeting the AhR-ROS-autophagy axis (e.g., AhR antagonists, ROS scavengers, autophagy inducers) can inhibit CS-induced pulmonary cellular senescence and may serve as a promising therapeutic strategy for COPD.
